# Multiomics Approach Distinguishes SPTBN4 as a Key Molecule in Diagnosis, Prognosis, and Immune Suppression of Testicular Seminomas

**DOI:** 10.1155/ijog/3530098

**Published:** 2025-04-25

**Authors:** Jianfeng Xiang, Yanjie Xiang, Qintao Ge, Yunhong Zhou, Hailiang Zhang, Wenhao Xu, Shifang Zhou, Liang Chen

**Affiliations:** ^1^Department of Interventional Oncology, Renji Hospital, Shanghai Jiao Tong University School of Medicine, Shanghai, China; ^2^Center for Reproductive Medicine, Xinhua Hospital Affiliated to Shanghai Jiao Tong University School of Medicine, Shanghai, China; ^3^Department of Urology, Fudan University Shanghai Cancer Center, Fudan University, Shanghai, China; ^4^Shanghai Genitourinary Cancer Institute, Shanghai, China; ^5^Department of Radiology, Fudan University Shanghai Cancer Center, Fudan University, Shanghai, China; ^6^Department of Obstetrics and Gynecology, Zhongshan Hospital, Fudan University, Shanghai, China; ^7^Interventional Department, Shanghai Jiao Tong University Affiliated Sixth People's Hospital South Campus (Shanghai Fengxian District Central Hospital), Shanghai, China

**Keywords:** immune suppression, multiomics analysis, prognostic biomarker, single-cell RNA sequencing, SPTBN4, testicular seminomas, tumor microenvironment

## Abstract

**Background:** Testicular seminomas, a common germ cell tumor, poses clinical challenges due to its molecular heterogeneity and limited biomarkers for precise diagnosis and prognosis. Leveraging multiomics approaches enables the comprehensive dissection of tumor complexity and facilitates the identification of key molecules influencing disease progression and therapeutic response.

**Methods:** Single-cell RNA transcriptomic sequencing (scRNA-seq) was utilized to explore the cellular and transcriptional heterogeneity of testicular seminomas. High-dimensional weighted gene coexpression network analysis (hdWGCNA) identified gene modules linked to tumor progression. Public datasets were integrated for gene expression and survival analyses, and drug sensitivity patterns were assessed using the GDSC database.

**Results:** scRNA-seq analysis revealed heterogeneous epithelial populations, with Epi1 cells exhibiting SLC5A5 and SPTBN4 as risk factors for advanced progression of seminomas. hdWGCNA identified nine gene modules, with the M6 module significantly enriched in Epi1 cells, implicating pathways such as negative regulation of ERAD and selective mRNA degradation. SPTBN4 was markedly upregulated in seminoma compared to nonseminomatous tumors and normal tissues, and its high expression was associated with poorer clinical outcomes and immunosuppressive microenvironments. Immune pathway analyses highlighted reduced antigen presentation and increased neutrophil extracellular trap (NET) formation in the SPTBN4-high group, suggesting diminished immunotherapeutic efficacy. Conversely, the SPTBN4-high group exhibited increased sensitivity to multiple chemotherapeutic agents, including thapsigargin and sorafenib, indicating its potential as a predictive marker for chemotherapy.

**Conclusion:** In conclusion, this multiomics study identifies SPTBN4 as a central biomarker in testicular seminomas, encompassing diagnostic, prognostic, and therapeutic dimensions. The integration of single-cell transcriptomics, hdWGCNA, and drug sensitivity analyses underscores the molecular complexity of seminomas and highlights the translational potential of SPTBN4 in guiding personalized treatment strategies. These findings provide a foundation for leveraging multiomics approaches to advance the clinical management of testicular seminomas and other heterogeneous malignancies.

## 1. Introduction

Testicular cancer (TCGT) is one of the most common malignancies among young men, with a steadily increasing incidence globally, posing a significant threat to male reproductive health and quality of life [1]. Each year, more than 70,000 new cases of testicular cancer are diagnosed worldwide, and its incidence has nearly doubled over the past 50 years, particularly among Caucasian populations [2, 3]. Epidemiological data indicate that testicular cancer predominantly affects males aged 15–35 years, with approximately 95% of cases classified as germ cell tumors (GCTs) [4]. Seminoma, a major subtype of testicular GCTs, exhibits unique clinicopathological characteristics and biological behavior [4, 5]. While seminomas are typically highly responsive to radiotherapy and chemotherapy, with a 5-year overall survival rate exceeding 95%, approximately 15% of patients experience relapse or disease progression after treatment, and the underlying molecular mechanisms remain poorly understood [6, 7].

Current diagnostic approaches for testicular cancer primarily rely on imaging studies and serum biomarkers, such as *α*-fetoprotein (AFP) and human chorionic gonadotropin (hCG) [8]. However, these traditional methods have limitations in specificity and sensitivity, particularly for early detection and accurate subtyping of seminomas. Moreover, despite the overall effectiveness of chemoradiation therapy, a subset of seminoma patients faces challenges such as treatment relapse and drug resistance [7]. These findings underscore the urgent need for molecular biomarkers that enhance diagnostic precision and prognostic prediction for this subtype.

Recent studies have further confirmed that testicular cancer development may involve multiple factors, including genetic susceptibility, environmental exposure, and abnormalities in reproductive system development [9]. For instance, genome-wide association studies in recent years have identified several genetic variants, such as KITLG, SPRY4, and DMRT1, as key contributors to seminoma susceptibility. However, these studies have yet to elucidate the specific molecular mechanisms involved. In general, there remains a pressing need to deepen research into the molecular characteristics of seminomas and their clinical applications in precision medicine.

The tumor microenvironment (TME), comprising tumor cells, immune cells, fibroblasts, vascular endothelial cells, and extracellular matrix components, plays a critical role in regulating tumor initiation, progression, and therapeutic responses [10, 11]. Dynamic changes in the TME significantly influence tumor cell proliferation, invasion, and metastasis while modulating treatment sensitivity. Studies have demonstrated that the immune components of the TME in seminomas are closely associated with tumor progression and patient prognosis [12–14]. For example, the abundance of tumor-infiltrating lymphocytes (TILs) has been positively correlated with favorable outcomes, whereas an increase in immunosuppressive cells, such as regulatory T cells (Tregs) and myeloid-derived suppressor cells (MDSCs), may accelerate disease progression by promoting immune evasion and resistance in testicular cancer.

In recent years, the rapid development of single-cell RNA sequencing (scRNA-seq) has provided powerful tools for dissecting the heterogeneity of the TME. Unlike traditional bulk transcriptomics, scRNA-seq enables the identification of transcriptional characteristics at single-cell resolution, unveiling the functional states and interaction patterns of various cell types within tumors and their microenvironments [15]. In seminomas, this technology offers a novel perspective for identifying specific cellular subsets and their molecular features within the TME [16, 17]. However, despite the significant advancements achieved in other solid tumors, systematic studies focusing on testicular cancer, particularly seminomas, remain limited [18]. Comprehensive characterization of the cellular composition and functional properties of the seminoma TME is critical for elucidating tumor progression mechanisms and identifying potential therapeutic targets.

Although existing studies have preliminarily highlighted the importance of the TME in testicular cancer progression, significant gaps remain at the molecular level, especially regarding the specific features of different subtypes, such as seminomas [19]. On the one hand, current diagnostic markers for seminomas are primarily confined to histological and serum-level markers, lacking specificity to distinguish seminomas from other testicular cancer subtypes. On the other hand, prognostic studies on seminomas have yet to establish unified molecular indicators, particularly for assessing patient responses to immunotherapy [20].

In recent years, the successful application of immune checkpoint inhibitors in various malignancies has offered new hope for cancer immunotherapy [13]. However, in seminomas, the efficacy of immunotherapy shows significant variability, likely due to the immunosuppressive cells and molecular networks within the TME [21, 22]. Furthermore, most studies have focused on specific genes or signaling pathways without adopting systematic and integrative research strategies [23–25]. Thus, leveraging single-cell transcriptomics in conjunction with large-scale transcriptomic databases to comprehensively characterize the molecular features of the seminoma TME and identify reliable diagnostic and prognostic markers is of substantial research value.

Based on the aforementioned background, this study is aimed at analyzing the cellular composition and functional characteristics of the testicular cancer TME through single-cell transcriptomics and integrating findings with large-scale transcriptomic databases to systematically identify molecular features associated with testicular seminomas. The primary discovery of this study is the identification of SPTBN4 as a critical molecular marker for testicular seminomas. Its high expression not only facilitates precise diagnosis of this subtype but also indicates poor prognosis and significant immunosuppressive features within the TME. Furthermore, elevated SPTBN4 expression is closely associated with immune evasion mechanisms and poor immunotherapy outcomes. Through this study, we aim to provide critical scientific evidence for the basic research and clinical application of testicular cancer, particularly seminomas, and drive further advancements in this field.

## 2. Methods and Materials

### 2.1. scRNA-seq Data Collection and Preprocessing

The single-cell transcriptome sequencing data (scRNA) and corresponding clinical profiles were retrieved from the Gene Expression Omnibus (GEO, https://www.ncbi.nlm.nih.gov/geo/). GSE197778 contained four samples from the single individual, including primary testicular seminoma tissues, pelvic lymph node, renal hilus lymph node, and peripheral blood. As the metastatic tumor was solely found in pelvic lymph node [26], only testicular seminoma tissues and pelvic lymph node were used for further study.

Using the R package DoubletFinder [27], doublets were removed and low-quality cells were filtered out. The exclusion criteria were cells with a gene count exceeding 6000 or below 200 and cells with a mitochondrial gene count percentage greater than 10%. During the gene filtering step, any gene expressed in fewer than five cells was excluded. The CellCycleScoring() function was employed to evaluate cell cycle states, and a regression algorithm was applied in the ScaleData() function to mitigate the impact of cell cycle variation. After normalizing and scaling the expression data, batch effects were addressed using the R package Harmony, which also facilitated the selection of the top 2000 variable genes. Dimensionality reduction was performed through principal component analysis (PCA) based on these 2000 variable genes, with the distances within the top 15 principal components used to generate Uniform Manifold Approximation and Projection (UMAP) embeddings. Cell clustering was conducted using the FindClusters() function at a resolution of 0.4. Clusters were manually annotated by referencing marker genes associated with various cell types.

### 2.2. Animal Models

Male BALB/c nude mice (4-week-old, SPF grade) were purchased from Shanghai Jihui Laboratory Animal Co. Ltd.; 2–5 × 10^5^ (100 *μ*L) luciferase-labeled cells H2030-BrM5-Luciferase and H2030-BrM5-shSEC61G-Luciferase were inoculated in eight mice. For brain metastasis models, mice were injected with 1 × 10^5^ tumor cells via the carotid artery. Animals were monitored for neurological symptoms and sacrificed at ethical endpoints. Tumor tissues were collected for histopathology and immunohistochemical (IHC) analysis. All animal experiments were approved by the Ethics Committee of Fudan University Cancer Hospital and conducted in accordance with institutional guidelines.

### 2.3. Collection and Preprocessing of Bulk Sequencing Data

Information regarding gene expression and detailed clinical data for individuals diagnosed with TCGT was obtained from The Cancer Genome Atlas (TCGA, https://portal.gdc.cancer.gov/). We included a total of 132 samples from the TCGA-TGCT cohort, which consisted of 62 seminomas, 62 nonseminomas, and 8 mixed tumor cases. Gene sequencing results for the four groups were expressed in transcripts per million (TPM). To enable effective comparison, the original expression data from the TCGA-TGCT was transformed to log2(TPM + 1).

### 2.4. High-Dimensional Weighted Gene Coexpression Network Analysis (hdWGCNA)

We conducted hdWGCNA to pinpoint crucial genes linked to different cell types [28]. scRNA data allowed for the selection of specific populations, followed by the construction of a gene expression correlation matrix, development of weighted gene coexpression networks, and detection of modules. The analysis of module–trait relationships revealed modules that were significantly correlated with the metastatic cell types, while hub genes within these notable modules were identified through their connectivity within the modules. The leading 25 hub genes were recognized as key genes.

### 2.5. Functional Enrichment Analysis and GSEA

The identification of differentially expressed genes (DEGs) across the groups was performed using the “limma” package. The criteria for filtering DEGs involved a minimum absolute fold change of greater than 1 and a *p* adjusted value of below 0.05. For the enrichment analyses pertaining to Gene Ontology (GO), Kyoto Encyclopedia of Genes and Genomes (KEGG), and HALLMARK, the R package “org.Hs.eg.db” [29] and “clusterProfiler” were utilized. GSEA was further conducted and a nominal *p* value < 0.05 and a false discovery rate (FDR) *q* value < 0.25 were used as cutoffs for significance. The R package “GseaVis” was used.

### 2.6. Univariate Cox Regression and Pan-Cancer Analyses

Univariate Cox regression analysis, along with pan-cancer analysis, was performed to assess the gene expression profiles. The gene expression data for pan-cancer were sourced from UCSC Xena (https://xena.ucsc.edu/). We further refined the expression levels of 35 genes, which included the top 10 DEGs from metastatic tumor cells and the top 25 coexpressed genes identified via hdWGCNA, utilizing univariate Cox regression analysis. A hazard ratio (HR) exceeding 1 with a *p* value below 0.05 was considered a risk factor, whereas an HR below 1 with a *p* value under 0.05 suggested a protective factor. Furthermore, we analyzed the filtered genes in comparison to normal tissues against tumor samples across 34 different tumor types.

### 2.7. CIBERSORT Deconvolution Analysis

To assess the fraction of immune cell types within the sample, we employed the CIBERSORT tool (https://cibersort.stanford.edu/), which utilizes a weighted least squares support vector regression (SVR) methodology to examine the composition of immune cells derived from mixed transcriptomic datasets. For the purpose of this study, we chose the LM22 gene matrix, which encompasses gene expression profiles pertinent to 22 distinct immune cell varieties. The input data was modified to satisfy the criteria necessary for CIBERSORT analysis. Initially, we transformed raw RNA-Seq or microarray data into a normalized gene expression matrix, which was then processed through the CIBERSORT algorithm. The analysis was conducted using standard parameters and repeated 1000 times to bolster the robustness of the findings. Samples with a *p* value less than 0.05, as indicated by CIBERSORT, were considered for further examination to guarantee the precise inference of immune cell composition. Ultimately, we acquired the relative distributions of the 22 immune cell subtypes for each sample. The outcomes from this analysis were subsequently employed in association studies, assessments of group distinctions, and a thorough examination of immune signatures.

### 2.8. Comparation of Immunotherapy Efficacy and Chemotherapy

Of published studies, we identified 24 molecular entities associated with immunotherapy inhibition, which were subsequently utilized for the Spearman correlation analysis [30, 31]. Additionally, we gathered the tumor immune dysfunction and exclusion (TIDE) scores for TGCT cases directly from the TIDE website (https://tide.dfci.harvard.edu/), which allowed us to assess the likelihood of immune evasion and the potential advantages of immunotherapy. Furthermore, we utilized the SubMap module within GenePattern (https://cloud.genepattern.org/) to predict responses to anti-PD-1 and anti-CTLA4 therapy.

To further evaluate the different responses of patients to chemotherapy, we obtained data from GDSC2016 (https://www.cancerrxgene.org/) to enter into the ComDrug program in “MOVICS” package [32]. The estimated inhibitory concentration (IC50) was calculated for each patient via ridge regression analysis to represent their response to different drugs.

### 2.9. IHC Staining Analysis

Tumor tissues were collected for histopathology and IHC analysis. All experiments were approved by the Ethics Committee of Fudan University Cancer Hospital and conducted in accordance with institutional guidelines (No. FUSCC-IACUC-S20210510, FUSCC, Shanghai, China). Six individual samples representing three different tissue types were used: adjacent normal tissue (*N* = 3), primary tumor (*N* = 3), and lymph node metastasis (*N* = 3). The research included tissue microarrays sourced from patients who had undergone radical orchiectomy. We performed IHC staining to analyze the expression of SPTBN4, utilizing an anti-SPTBN4 antibody (Cat. PA5-27088, Thermo Fisher, United States). The specific IHC protocol follows the procedures described in our earlier research [24]. Specimens were gathered and maintained in a 4% formaldehyde solution for 24 h, subsequently embedded in paraffin and cut into about 5-*μ*m thick sections. The tumor sections underwent deparaffinization and rehydration, followed by inhibiting endogenous peroxidase activity and retrieving antigens. Afterward, a 5% BSA solution was administered to minimize nonspecific binding for 30 min, and the sections were then incubated overnight with the primary antibody. Following a 1-h exposure to the secondary antibody, visualization of the tumor sections was accomplished using a DAB kit.

### 2.10. Statistical Analysis

The examination of categorical data was conducted through Fisher's exact test as well as the rank sum test. When comparing two groups, a *T*-test was utilized, while ANOVA enabled pairwise comparisons across multiple groups. Survival analysis was carried out using K-M curves. All statistical evaluations were executed with R (Version 4.2.2) or GraphPad Prism 9.0 software. A two-tailed *p* value of less than 0.05 was deemed statistically significant.

## 3. Results

### 3.1. Distinct Cellular Heterogeneity in Primary and Metastatic Seminomas

To elucidate the cellular heterogeneity between primary and metastatic seminomas, scRNA-seq was performed on testicular and lymph node tissues (Supporting Information 1: Figure S1A). After stringent quality control, 10,395 cells were retained and grouped into 16 distinct clusters (Supporting Information 1: Figure S1B), with 13 major cell populations successfully annotated based on established markers.

**Figure 1:**
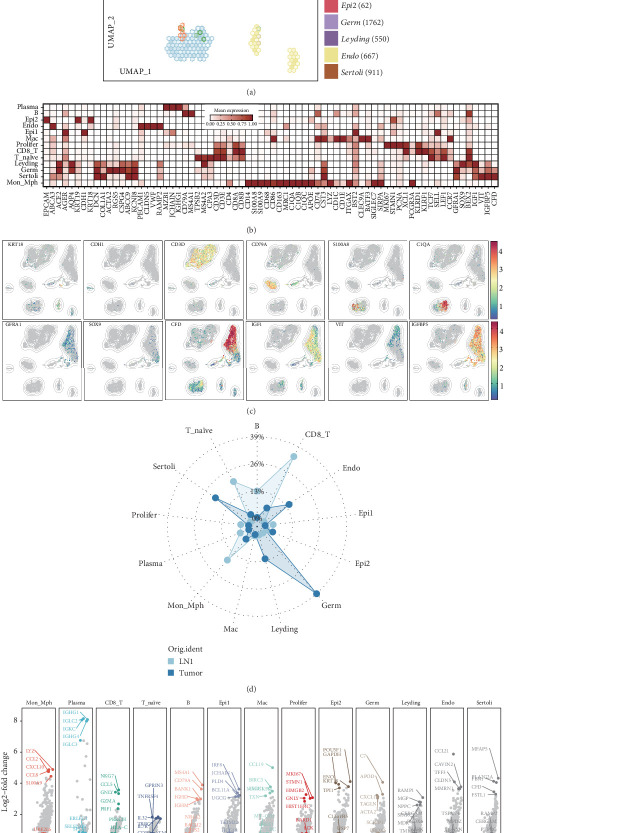
Distinct cellular heterogeneity in primary and metastatic testicular seminomas. (a) Uniform Manifold Approximation and Projection (UMAP) visualization of 16 distinct cell clusters identified in testicular and lymph node seminoma tissues, showcasing 10 prominent cell populations. (b) Heatmap showing the expression of key markers for various cell populations in seminoma, including immune cells and epithelial cells, as well as other cell types such as germ cells and Sertoli cells. (c) Expression distribution of cell-specific markers across the cell populations. (d) Comparative analysis of immune cell composition between primary testicular seminomas and lymph node metastases, highlighting the enrichment of T cells and macrophages in metastatic lymph nodes. (e) Transcriptional differences between Epi1 and Epi2 subtypes, with notable upregulation of IRF8 and JCHAIN in Epi1 and POU5F1 and ENO1 in Epi2, indicating distinct functional roles in tumor progression.

UMAP visualization of 10 distinct cell populations is shown in Figure 1a. Among the immune cell populations, monocytes/macrophages (Mon/Mph) (1222 cells) were characterized by the expression of classical markers including CD14, S100A8, S100A9, CD83, CD163, C1QB, and C1QA. Plasma cells (296 cells) exhibited high expression of MZB1, IGHG1, and CD79A, while B cells (796 cells) prominently expressed CD79A. Notably, CD8+ T cells (2283 cells) displayed elevated expression of CD3D, CD3E, CD8A, and CD8B, whereas naïve T cells (1326 cells) were defined by SELL, CCR7, CD3D, and CD3E. Epithelial cell populations included two transcriptionally distinct subsets: Epi1 (228 cells) and Epi2, both expressing markers such as EPCAM, KRT18, and AQP4. However, Epi1 and Epi2 showed distinct transcriptional profiles. Macrophages (46 cells) were identified by the unique expression of macrophage-related markers such as CD86 and C1QA. Proliferating cells (246 cells) expressed CD3D, CD3E, XCL1, and KLRF1. Endothelial cells (667 cells) were characterized by high expression of VWF and CLDN5, while Leydig cells (550 cells) expressed markers like IGF1, VIT, IGFBP4, and CFD. Germ cells (1762 cells) exhibited GFRA1 expression, and Sertoli cells (911 cells) were identified by the expression of SOX9 and BEX2 (Figure 1b,c).

A comparative analysis revealed distinct immune cell compositions between testicular tissues and lymph node metastases, with lymph nodes showing an enrichment of T cells and macrophages (Figure 1d). Interestingly, the epithelial populations exhibited site-specific dominance, with Epi1 constituting the majority in metastatic lymph nodes, whereas Epi2 was predominant in primary testicular tumors. Transcriptional profiling highlighted that Epi1 was characterized by elevated expression of IRF8, JCHAIN, PLD4, BCL11A, and UGCG, while Epi2 exhibited higher levels of POU5F1, ENO1, KRT18, and TPI1 (Figure 1e and Supporting Information 1: Figure S1C). These findings underscore the presence of distinct epithelial subpopulations with unique transcriptional profiles, suggesting that Epi1 may represent a malignant epithelial lineage associated with tumor progression and immune evasion in seminomas.

### 3.2. Identification of Key Gene Modules and Functional Pathways in Progression-Associated Tumor Cells via hdWGCNA

**Figure 2:**
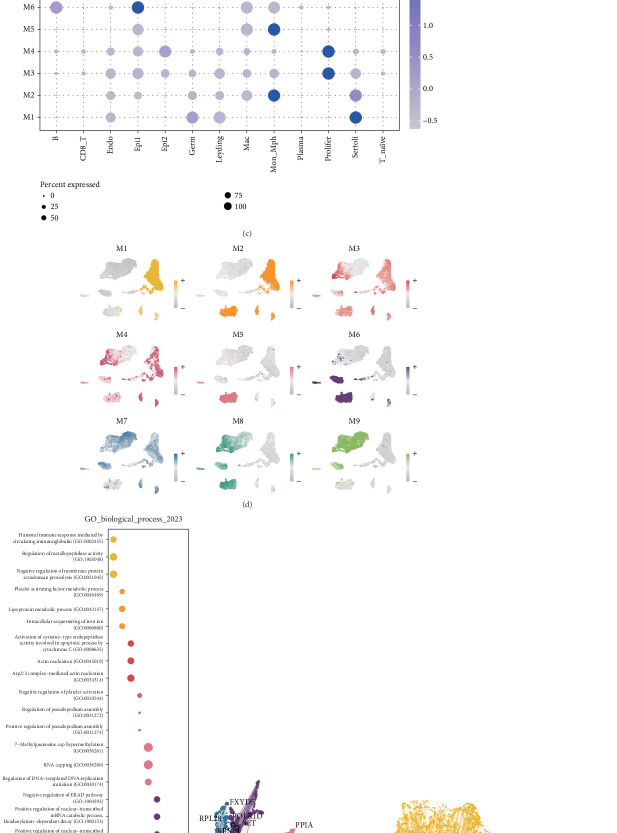
Identification of key gene modules and functional pathways in progression-associated tumor cells via high-dimensional weighted gene coexpression network analysis (hdWGCNA). (a) Weighted gene coexpression network construction (hdWGCNA) showing the optimal soft threshold power (*β* = 8) selected to achieve a scale-free topology index of 0.90. (b) Cluster dendrogram of the nine gene modules identified, each representing distinct biological processes potentially involved in seminoma progression. (c) UMAP visualization of the expression pattern of the M6 module in Epi1 cells, highlighting its specific activation. (d) Heatmap illustrating the top 25 genes in the M6 module, including SET, FXYD5, and SPTBN4, with a focus on their elevated expression in Epi1 cells. (e) Functional enrichment analysis of the M6 module, showing significant involvement in pathways such as ERAD pathway suppression and mRNA tail shortening, both implicated in tumor progression. (f) Gene–gene interaction network for the M6 module, illustrating the interconnectedness of the identified key genes and their functional implications in seminoma progression. (g) The top 25 coexpressed genes in the M6 module.

To delineate the molecular underpinnings of progression-associated tumor cells, we applied hdWGCNA. During network construction, the soft threshold power (*β* = 8) was selected to achieve a scale-free topology fitness index of 0.90, ensuring an optimally connected, unweighted cell network (Figure 2a). This approach identified nine distinct gene modules (Figure 2b), each representing a cohesive set of coexpressed genes with potential functional relevance to tumor progression.

Among these, the M6 module demonstrated specific and pronounced activation in Epi1 cells (Figure 2c,d), positioning it as the module most strongly associated with this cell population. Functional enrichment analysis of the M6 module revealed significant involvement in diverse signaling pathways, including the negative regulation of the endoplasmic reticulum–associated degradation (ERAD) pathway and the positive regulation of nuclear-transcribed mRNA tail shortening (Figure 2e). These findings suggest that Epi1 cells may enhance their growth capacity by suppressing the ERAD pathway, thereby stabilizing oncogenic proteins, while simultaneously promoting mRNA tail shortening to selectively degrade transcripts of tumor suppressor genes.

The coexpression network further illustrated the intricate gene–gene interactions within each module, highlighting the functional connectivity underpinning these genes (Figure 2f). For in-depth analysis, the top 25 coexpressed genes in each module were visualized (Supporting Information 2: Figure S2), with particular focus on the M6 module. Key genes in the M6 module, including SET, FXYD5, SLC5A5, SPTBN4, and NCL, were identified as pivotal contributors to the aggressive phenotype of Epi1 cells and were selected for further experimental validation (Figure 2g). These insights provide a robust molecular framework for understanding the aggressive behavior of progression-related tumor cells and highlight potential therapeutic targets within the M6 module.

### 3.3. SPTBN4 Serves as a Novel Biomarker for Seminoma and Poor Prognosis

To identify potential biomarkers associated with seminoma and its prognosis, we integrated DEGs from Epi1 cells, hdWGCNA modules, and the TCGA-TCCT dataset. This analysis yielded 33 intersecting genes, which were subsequently subjected to univariate Cox regression analysis. Among these, SLC5A5 and SPTBN4 emerged as risk factors for seminoma, whereas PLD4 was identified as a protective factor. The remaining genes did not exhibit significant prognostic relevance (Table 1).

**Figure 3:**
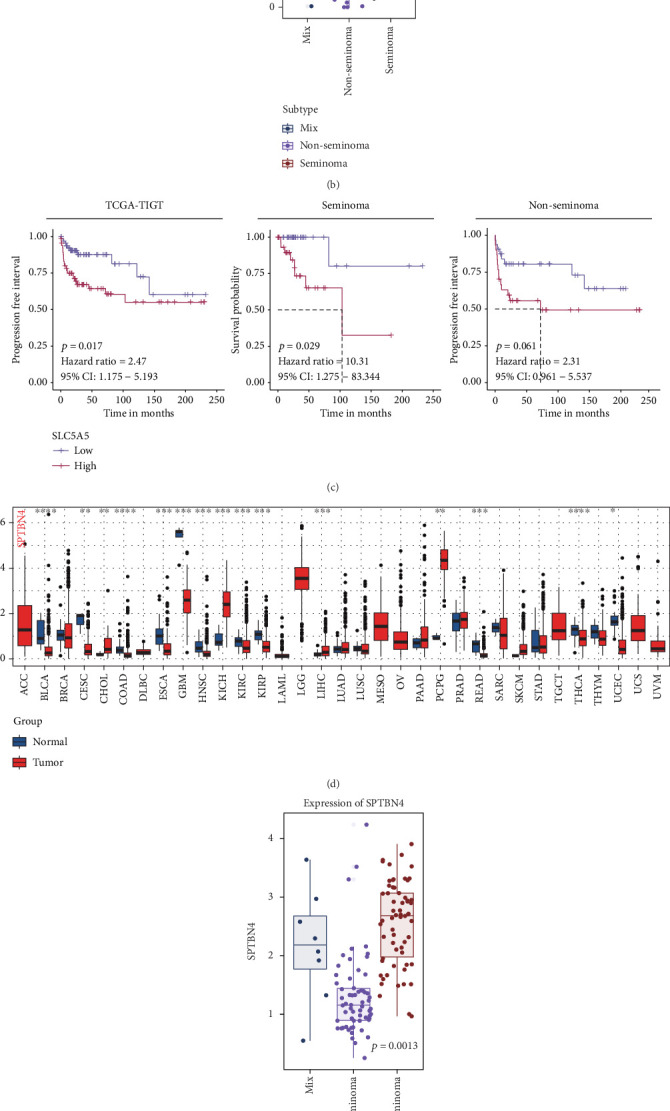
SPTBN4 as a novel biomarker for seminoma and poor prognosis. (a) Pan-cancer analysis of SLC5A5 expression across 34 cancer types, highlighting its upregulation in testicular tumors, particularly in nonseminomatous tumors. (b) Subtype-specific expression of SLC5A5 in testicular tumors, with increased expression in nonseminomatous tumors compared to seminomas. (c) Survival analysis showing the association between high SLC5A5 expression and increased risk of recurrence in seminoma patients. (d) Expression comparison of SPTBN4 across seminomas and nonseminomatous tumors, demonstrating significantly higher levels in seminomas. (e) Kaplan–Meier survival analysis for SPTBN4 expression in seminoma patients, showing a significant association with poor prognosis. (f) Prognostic analysis of SPTBN4 expression in testicular cancer subtypes, indicating its unique prognostic value in seminomas.

Pan-cancer analysis of SLC5A5 across 34 cancer types revealed distinct expression patterns (Figure 3a). While SLC5A5 expression was generally low across various malignancies, it was markedly upregulated in testicular tumors, underscoring its potential as a tumor-specific marker. Subtype analysis further demonstrated higher SLC5A5 expression in nonseminomatous tumors compared to mixed and seminomatous tumors (Figure 3b, *p* < 0.030).

Survival analysis revealed a significant association between SLC5A5 expression and recurrence risk in testicular cancer patients (*p* = 0.017, HR = 2.47, 95% CI: 1.175–5.193). Seminoma patients with high SLC5A5 expression had a markedly increased risk of recurrence, with an approximately tenfold greater risk compared to those with low expression (*p* = 0.032, HR = 10.31, 95% CI: 1.275–83.344). Similarly, nonseminomatous tumor patients with high SLC5A5 expression exhibited a twofold increased recurrence risk (*p* = 0.061, HR = 2.31, 95% CI: 0.961–5.537) (Figure 3c). These findings suggest that SLC5A5 may serve as a prognostic indicator, particularly in seminoma. SPTBN4 was identified as uniquely expressed in testicular tumor tissues, with significantly higher levels in seminoma compared to nonseminomatous and mixed tumors (Figure 3d,e, *p* = 0.0013). Further IHC analysis confirmed that SPTBN4 presented elevated expression in lymph node metastatic tumor compared to adjacent and primary tumors (Supporting Information 3: Figure S3). While SPTBN4 did not demonstrate prognostic significance in normal testicular tissues (*p* = 0.496, HR = 0.78, 95% CI: 0.39–1.578), subgroup analysis revealed that high SPTBN4 expression was associated with poorer prognosis in seminoma patients (*p* = 0.032, HR = 9.86, 95% CI: 1.217–79.87). No significant relationship was observed in nonseminomatous tumors (*p* = 0.889, HR = 1.06, 95% CI: 0.459–2.453) (Figure 3f).

In addition to SPTBN4, 31 other molecules were analyzed, revealing potential testicular tumor markers such as TPI1, HMGN1, UGCG, NCL, SET, and EIF3K (Supporting Information 4: Figure S4A). These molecules were significantly upregulated in testicular cancers and could enhance diagnostic precision. However, their expression differences among the three tumor subtypes were minimal, with predominant expression observed in nonseminomatous tumors (Supporting Information 4: Figure S4B).

Our findings highlight SPTBN4 as a promising biomarker for seminoma diagnosis and prognosis. Its unique expression in seminoma and its association with poor prognosis underscore its potential clinical utility. Additionally, the identification of SLC5A5 and other candidate markers provides a broader framework for understanding testicular cancer heterogeneity and developing targeted diagnostic strategies.

### 3.4. High Expression of SPTBN4 in Seminoma Correlates With Immune Suppression and Neutrophil Extracellular Trap (NET) Formation

**Figure 4:**
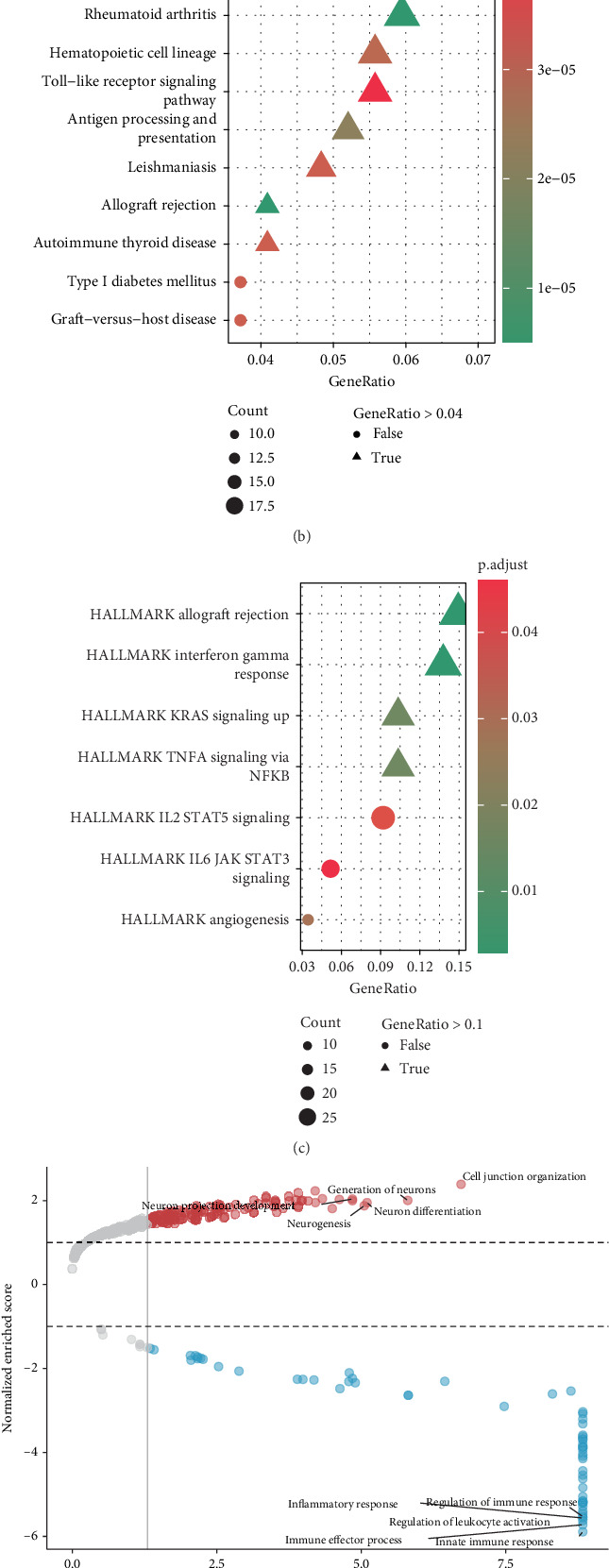
High expression of SPTBN4 in seminoma correlates with immune suppression and neutrophil extracellular trap (NET) formation. (a) Gene Ontology (GO) enrichment analysis comparing high and low SPTBN4 expression groups, highlighting immune evasion pathways such as antigen processing, cell–cell interactions, and immune suppression. (b) KEGG pathway enrichment showing the activation of immune suppression–related pathways in the high SPTBN4 expression group, including cell adhesion molecules and Toll-like receptor signaling. (c) HALLMARK pathway enrichment analysis revealing the association of high SPTBN4 expression with pathways like allograft rejection and interferon gamma response. (d) Gene set enrichment analysis (GSEA) based on the Gene Ontology biological process (BP) database showing upregulation of cell junction and neurogenesis pathways in the high-expression group, contrasting with stronger immune activation in the low-expression group. (e) KEGG-based GSEA revealing significant enrichment of NET formation pathways in the high SPTBN4 expression group, highlighting its potential role in immune suppression and poor immunotherapy outcomes.

To explore the functional implications of SPTBN4 expression in seminoma, we conducted pathway enrichment analyses comparing groups with high and low SPTBN4 expression levels. GO analysis revealed distinct enrichment patterns between the two groups. In the high SPTBN4 expression group, significant pathways included antigen processing and peptide antigen presentation, as well as the regulation of cell–cell interactions, emphasizing the involvement of SPTBN4 in immune evasion mechanisms (Figure 4a). Immune-related processes, such as adhesion and cellular communication, also exhibited notable differences, suggesting a pivotal role for SPTBN4 in tumor–immune microenvironment interactions.

KEGG pathway analysis identified significant enrichment of pathways such as cell adhesion molecules, Toll-like receptor signaling, antigen processing and presentation, and autoimmune thyroid disease in the high SPTBN4 expression group (Figure 4b). These pathways are known to influence tumor immune suppression and progression, further substantiating the potential role of SPTBN4 in immune modulation. Using the HALLMARK database, pathways such as HALLMARK allograft rejection, HALLMARK interferon gamma response, and HALLMARK KRAS signaling up were significantly enriched in the high SPTBN4 expression group (Figure 4c). GSEA based on the GO biological process (BP) database revealed upregulation of pathways associated with cell junction organization and neurogenesis in the high-expression group. Conversely, pathways linked to immune response regulation, immune effector processes, and innate immune responses were more active in the low SPTBN4 expression group, indicating a more robust immune environment in these tumors (Figure 4d).

KEGG-based GSEA provided further insights into pathway activation. High SPTBN4 expression was significantly associated with increased NET formation, a process implicated in immune suppression and poor outcomes in cancer immunotherapy [33, 34]. In contrast, the low SPTBN4 expression group exhibited enhanced activation of the PI3K-Akt and AMPK signaling pathways, which are linked to cellular energy metabolism and survival (Figure 4e). These findings underscore the critical role of SPTBN4 in shaping the immune landscape of seminoma. Elevated SPTBN4 expression is associated with immune suppression pathways, including NET formation, which may contribute to reduced efficacy of immunotherapy. By contrast, tumors with low SPTBN4 expression display a more active immune profile and signaling pathways conducive to tumor suppression.

### 3.5. High Expression of SPTBN4 in Seminoma Predicts Poor Response to Immunotherapy

**Figure 5:**
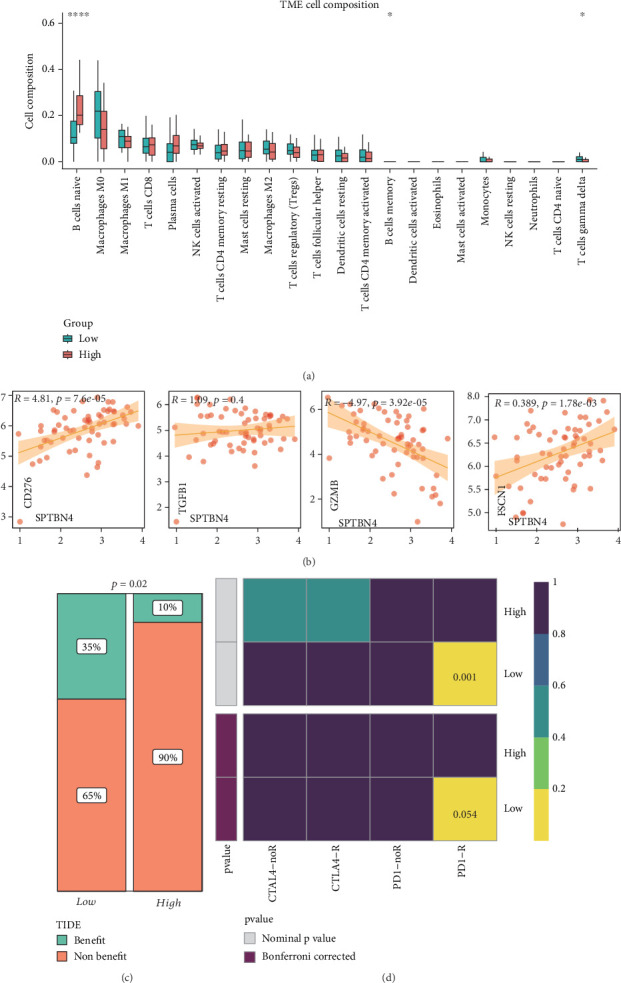
High expression of SPTBN4 in seminoma predicts poor response to immunotherapy. (a) Immune cell infiltration analysis using the CIBERSORT algorithm, comparing immune profiles between high and low SPTBN4 expression groups. The high-expression group exhibits reduced immune cell presence, particularly M0 and M1 macrophages. (b) Correlation analysis between SPTBN4 expression and immunosuppressive markers (e.g., CD276, TGFB1, FSCN1), showing a positive association with immune evasion markers and negative correlation with cytotoxic markers like GZMB. (c) TIDE analysis predicting immunotherapy response, demonstrating a significantly higher proportion of patients in the low SPTBN4 expression group likely to benefit from anti-PD-1 therapy (*p* = 0.02). (d) SubMap analysis confirming the increased sensitivity of patients with low SPTBN4 expression to anti-PD-1 treatment (*p* = 0.001, Bonferroni-corrected *p* = 0.054).

To elucidate the relationship between SPTBN4 expression, the immune microenvironment, and immunotherapy efficacy in seminoma, we utilized the CIBERSORT deconvolution algorithm to analyze immune cell infiltration. Results revealed that the SPTBN4 low-expression group displayed significantly greater infiltration of immune cells, particularly M0 and M1 macrophages, indicative of a proinflammatory immune microenvironment (Figure 5a). Conversely, the high-expression group exhibited reduced immune cell presence, consistent with an immunosuppressive phenotype.

Correlation analyses demonstrated that SPTBN4 expression was positively associated with immunosuppressive molecules such as CD276 (*R* = 4.81, *p* = 7.6 × 10^−5^), TGFB1 (*R* = 1.09, *p* = 0.4), and FSCN1 (*R* = 0.389, *p* = 1.78 × 10^−3^), while negatively correlated with the cytotoxic molecule GZMB (*R* = −4.97, *p* = 3.92 × 10^−5^) (Figure 5b). This suggests that elevated SPTBN4 expression contributes to the creation of an immunosuppressive TME.

To further assess immunotherapy responsiveness, TIDE analysis indicated that 35% of patients in the SPTBN4 low-expression group were likely to benefit from anti-PD-1 therapy, compared to only 10% in the high-expression group (*p* = 0.02) (Figure 5c). SubMap analysis supported this observation, demonstrating that patients with low SPTBN4 expression exhibited significantly increased sensitivity to anti-PD-1 treatment (*p* = 0.001, Bonferroni-corrected *p* = 0.054) (Figure 5d).

### 3.6. High Expression of SPTBN4 in Seminoma Indicates Favorable Response to Chemotherapy

**Figure 6:**
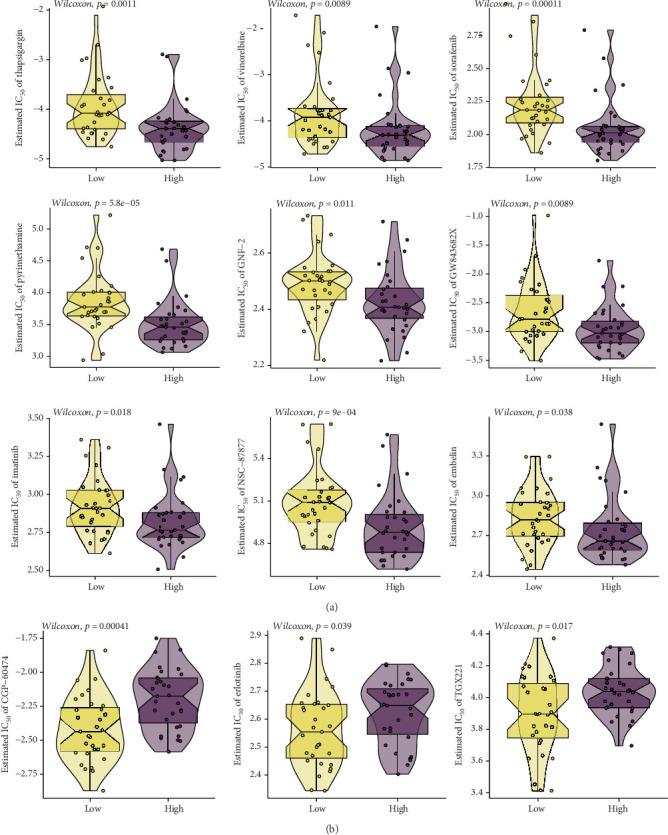
High expression of SPTBN4 in seminoma indicates favorable response to chemotherapy. (a) Drug sensitivity analysis using the GDSC database, showing a significantly higher sensitivity to various chemotherapeutic agents (e.g., thapsigargin, vinorelbine, sorafenib) in the high SPTBN4 expression group. (b) Sensitivity analysis of different chemotherapy agents in the low SPTBN4 expression group, revealing higher sensitivity to agents like CGP-60474 and erlotinib. These findings suggest that high SPTBN4 expression correlates with enhanced chemotherapeutic efficacy, positioning SPTBN4 as a predictive biomarker for chemotherapy response in seminoma.

Using the GDSC database, we evaluated drug sensitivity in seminoma patients stratified by SPTBN4 expression. The analysis identified 12 chemotherapeutic agents with significant differences in sensitivity, as measured by IC50 values. Patients in the high SPTBN4 expression group exhibited greater sensitivity to agents such as thapsigargin (*p* = 0.0011), vinorelbine (*p* = 0.0089), sorafenib (*p* = 0.00011), pyrimethamine (*p* = 5.8 × 10^−5^), GNF-2 (*p* = 0.011), GW843682X9 (*p* = 0.0089), imatinib (*p* = 0.018), NSC-87877 (*p* = 9 × 10^−4^), and embelin (*p* = 0.038) (Figure 6a).

In contrast, the low SPTBN4 expression group showed higher sensitivity to CGP-60474 (*p* = 0.00041), erlotinib (*p* = 0.039), and TGX221 (*p* = 0.017), suggesting potential utility of these agents for this subgroup. Overall, high SPTBN4 expression in seminoma was associated with a broader range of effective chemotherapeutic options, positioning these patients as favorable candidates for chemotherapy (Figure 6b). These findings delineate the dual role of SPTBN4 in seminoma therapy. High SPTBN4 expression correlates with an immunosuppressive microenvironment and diminished response to immunotherapy but predicts enhanced sensitivity to a wider spectrum of chemotherapeutic agents. This highlights SPTBN4 as a pivotal biomarker for guiding personalized treatment strategies in seminoma.

## 4. Discussion

SPTBN4 (spectrin beta nonerythrocytic 4) encodes a subunit of *β*-spectrin, a key component of the cytoskeleton involved in maintaining cell membrane stability, regulating cell morphology, facilitating cell migration, and mediating signal transduction [35]. It plays an essential role in various tissues, including the nervous system, muscles, and internal organs, where it provides structural support at the interface between the plasma membrane and the cytoskeleton [36]. Abnormalities in SPTBN4 expression or mutations in this gene have been associated with a range of diseases, particularly those affecting the nervous system, muscles, and metabolic processes [37]. These disruptions can lead to alterations in cell shape and impaired intercellular signaling, thereby impacting normal physiological functions. This study identified SPTBN4 as a pivotal molecular marker for seminomas, highlighting its dual role in precise diagnosis and prognostic prediction. The integration of single-cell and large-scale transcriptomics enabled comprehensive characterization of the seminoma TME. High SPTBN4 expression was not only associated with the immunosuppressive features of the TME but also correlated with poor clinical outcomes, including reduced responsiveness to immunotherapy.

In cancer, the aberrant expression and dysfunction of SPTBN family are implicated in tumor initiation, progression, and metastasis [38–40]. The protein regulates cytoskeletal remodeling, influencing critical processes such as tumor cell proliferation, migration, and invasion. Interestingly, SPTBN1 regulates the cytoplasmic constraint of pituitary tumor-transforming gene 1, promoting the invasive capability of human seminoma [41]. Studies have emphasized the effect and mechanism of tumor cell SPTBN1 on polarization and migration of tumor-associated macrophages in hepatoma and breast cancer, where its role is tightly linked to the biological behavior of tumors [42]. For instance, overexpression of SPTBN4 is associated with enhanced invasive potential, poor prognosis, and cisplatin resistance in lung cancer [43]. By affecting cell adhesion, motility, and cell cycle regulation, SPTBN4 may promote tumor cell growth and metastasis. Conversely, downregulation of SPTBN4 could influence tumor progression and vasculature, suggesting its potential as a diagnostic and a key regulator of tumor angiogenesis in cancer [44].

SPTBN4 also plays a significant role within the TME. The TME consists of tumor cells, immune cells, endothelial cells, and stromal components, whose interactions are crucial for tumor growth and metastasis [10]. SPTBN4 can influence tumor cell migration, immune evasion, and neovascularization within this context by mediating VEGFR2 internalization and degradation [44, 45]. For example, SPTBN4 modulates the interaction between tumor cells and the extracellular matrix, potentially facilitating matrix degradation and enhancing tumor invasiveness [45]. Furthermore, it may contribute to immune evasion mechanisms by altering cytoskeletal dynamics [46], thereby affecting immune cell recognition and elimination of tumor cells. As such, SPTBN4's involvement in the TME provides new avenues for research in tumor diagnosis and therapy, highlighting its potential as a therapeutic target.

The functional role of SPTBN4 in seminoma progression likely involves modulating immune evasion mechanisms and shaping an immunosuppressive microenvironment. Clinically, these findings suggest that SPTBN4 can serve as a diagnostic biomarker to differentiate seminomas from other testicular cancer subtypes. Additionally, the association between high SPTBN4 expression and poor prognosis underscores its potential as a prognostic marker. Importantly, the link between SPTBN4 and immunotherapy resistance highlights its utility in guiding personalized treatment strategies. Our findings align with prior research emphasizing the critical role of the TME in tumor progression and therapeutic resistance. For example, recent studies have demonstrated that seminoma subtypes exhibit distinct immune microenvironmental features. Less differentiated subtypes show significantly lower immune scores and reduced immune cell infiltration. Additionally, single-cell multiomics approaches have revealed unique cellular compositions and signaling pathways in seminomas. These observations underscore the value of our comprehensive approach, which situates SPTBN4 at the intersection of diagnostic, prognostic, and therapeutic landscapes [47].

This study's methodological strengths include the use of state-of-the-art single-cell transcriptomics to dissect the TME and the integration of large-scale transcriptomic datasets to validate findings. This approach provides a high-resolution analysis of the cellular composition within the tumor, allowing for the identification of distinct cell types and their specific roles, which traditional bulk RNA sequencing may not capture. Besides, the identification of SPTBN4 as a key molecular marker in testicular seminomas is a significant contribution. The study establishes SPTBN4 as a potential diagnostic biomarker for distinguishing seminoma subtypes, as well as a prognostic factor, offering a novel target for future therapeutic development. In addition, for the first time, this study links SPTBN4 overexpression with poor prognosis and immune-suppressive microenvironments, offering valuable insights into the immune landscape of testicular seminomas. This provides a foundation for future investigations into immune-based therapeutic strategies and immune checkpoint inhibitors for testicular cancer patients. These innovations pave the way for precision oncology approaches tailored to seminoma patients.

While this study provides robust evidence for the significance of SPTBN4, certain limitations must be acknowledged. First, the findings are primarily based on transcriptomic analyses, necessitating experimental validation through functional assays and clinical studies. Second, the study's focus on seminomas may limit generalizability to other testicular cancer subtypes. Future research should explore the mechanistic pathways involving SPTBN4 and assess its therapeutic potential in preclinical models [48]. Additionally, expanding the analysis to include longitudinal patient data could further elucidate its prognostic value.

## 5. Conclusion

In conclusion, this study establishes SPTBN4 as a key molecular marker for seminomas, bridging gaps in diagnosis, prognosis, and therapeutic response prediction. By advancing the understanding of seminoma biology and the TME, these findings contribute to the broader field of GCT research and support the development of targeted interventions. Ultimately, this work underscores the transformative potential of integrative omics approaches in shaping the future of precision oncology in testicular seminomas, offering a potential therapeutic target to enhance antitumor immunity.

## Figures and Tables

**Table 1:** Univariate Cox regression analysis for selected genes from single-cell analysis.

**Genes**	**HR**	**HR.95L**	**HR.95H**	**p** ** value**
TRAPPC6A	1.723327	0.429265	6.918471	0.442807662
HINT1	2.062886	0.513423	8.288496	0.307508762
LSM7	2.049707	0.510077	8.2366	0.311854981
EIF3K	3.189123	0.65415	15.54766	0.151325793
DHX36	1.647647	0.430714	6.302882	0.465712079
SYF2	0.41262	0.105052	1.620676	0.204719501
NCL	0.706302	0.188702	2.643651	0.605616495
HSP90AB1	2.40883	0.593458	9.777375	0.218712728
MAP4K4	0.934423	0.247712	3.524841	0.920242856
ARID4B	0.944765	0.253052	3.527264	0.932629658
UBXN1	2.574986	0.507919	13.05436	0.253444374
CHD7	0.926563	0.248106	3.460295	0.909669251
UBE2D2	1.927312	0.478791	7.758159	0.355783665
KMT2E	1.566174	0.414387	5.919351	0.508395058
ANP32B	1.25063	0.300938	5.197338	0.758298605
RPL22L1	1.601484	0.428685	5.982835	0.483722096
HMGN1	1.159247	0.302101	4.448362	0.829477398
MZT2A	3.125423	0.647837	15.07829	0.155814765
FXYD5	2.634554	0.5375	12.91326	0.232297607
SET	1.985888	0.492949	8.000327	0.334538285
POLR1D	1.371525	0.365422	5.147695	0.639670972
JCHAIN	0.426997	0.106573	1.710821	0.229484032
IRF8	1.052848	0.282197	3.928065	0.938893341
PLD4	0.198495	0.040581	0.970915	0.045889745
BCL11A	0.594419	0.153945	2.295188	0.45046088
TSPAN13	4.348592	0.867622	21.7955	0.073889476
TCL1A	1.000153	0.26277	3.80678	0.999821153
UGCG	1.385798	0.359313	5.344745	0.63567588
IRF7	0.192735	0.038527	0.964163	0.045027934
GPR183	0.772337	0.205466	2.903182	0.702179387
TXN	0.309021	0.07199	1.326492	0.114138407
SLC5A5	10.31023	1.275444	83.34426	0.028659558
SPTBN4	9.86154	1.217216	79.87352	0.032541102

## Data Availability

The data that support the findings of this study are available from the corresponding authors upon reasonable request.

## Supporting Information

Supporting Information 1 Figure S1. Cellular composition and transcriptional profiles of primary and metastatic seminomas. (a) Schematic overview of the study design, depicting the collection of testicular and lymph node tissues for single-cell RNA sequencing (scRNA-seq) analysis. (b) Uniform Manifold Approximation and Projection (UMAP) plot showing the clustering of 10,395 single cells into 16 distinct clusters. The clusters represent various cell populations identified based on established markers. (c) Transcriptional profiling of Epi1 and Epi2 populations. Heatmap showing the differential expression of key genes, including IRF8, JCHAIN, PLD4, BCL11A, UGCG (Epi1) and POU5F1, ENO1, KRT18, and TPI1 (Epi2), highlighting the distinct transcriptional signatures of these epithelial subsets in primary and metastatic seminomas.

Supporting Information 2 Figure S2. Top 25 coexpressed genes across WGCNA modules. Heatmaps showing the top 25 most coexpressed genes within each identified gene module from the high-dimensional weighted gene coexpression network analysis (hdWGCNA). Focus is placed on the M6 module, which is most strongly associated with the Epi1 population. Genes such as SET, FXYD5, SLC5A5, SPTBN4, and NCL are shown to contribute to the aggressive phenotype of Epi1 cells. The heatmaps represent the normalized expression levels of these genes across the tumor samples.

Supporting Information 3 Figure S3. SPTBN4 expression in lymph node metastatic tumors compared to adjacent and primary tumors. Immunohistochemical (IHC) analysis was conducted to assess the expression of SPTBN4 in tumor samples alongside adjacent normal tissue and primary tumors.

Supporting Information 4 Figure S4. Expression profiles of candidate biomarkers in testicular cancer subtypes. (a) Heatmap depicting the differential expression of 32 candidate biomarkers, including TPI1, HMGN1, UGCG, NCL, SET, and EIF3K, in testicular cancers. These genes were found to be significantly upregulated across testicular cancer types, with notable expression patterns observed in seminomas. (b) Expression comparison of candidate biomarkers among nonseminomatous, seminomatous, and mixed testicular tumors. The data suggest that these molecules are predominantly expressed in nonseminomatous tumors, with minimal differences observed across the three subtypes. ⁣^∗^*p* < 0.05; ⁣^∗∗^*p* < 0.01; ⁣^∗∗∗^*p* < 0.001; ⁣^∗∗∗∗^*p* < 0.0001.
